# Redo surgical aortic valve replacement versus valve-in-valve transcatheter aortic valve replacement with balloon valve fracture: Short-term clinical outcomes

**DOI:** 10.1016/j.xjse.2026.100127

**Published:** 2026-05-03

**Authors:** Rahul Kanade, Marco Tagliafierro, Isao Anzai, Fady Soliman, William M. Mitchell, Darina Kirilina, Paul Kurlansky, Rebecca T. Hahn, Susheel Kodali, Tamim M. Nazif, Torsten P. Vahl, Arnar Geirsson, Michael Argenziano, Isaac George, Luigi Pirelli

**Affiliations:** a Division of Cardiothoracic Surgery, Yale New Haven Hospital, New Haven, Conn; b Division of Cardiothoracic Surgery, Columbia University Irving Medical Center, New York-Presbyterian Hospital, New York, NY

**Keywords:** aortic valve, transcatheter valve-in-valve, bioprosthetic valve fracture, surgical valve replacement

## Abstract

**Objective:**

To evaluate short-term outcomes of redo surgical aortic valve replacement (SAVR) versus valve-in-valve transcatheter aortic valve replacement (ViV-TAVR) with bioprosthetic valve fracture (ViVBVF) in patients with bioprosthetic structural valve deterioration undergoing reintervention.

**Methods:**

This retrospective single-center study was conducted between 2015 and 2025. Data were obtained from institutional registries. A total of 128 consecutive patients met the inclusion criteria (75 with redo-SAVR and 53 with ViVBVF). Patients requiring concomitant cardiac procedures, mechanical valves, or unsuitable anatomy for ViV were excluded. The primary endpoints were 30-day mortality, stroke, and readmission; secondary endpoints included echocardiographic changes and postoperative complications. Overlap propensity score weighting to adjust baseline differences was performed.

**Results:**

Compared to the redo-SAVR patients, the ViVBVF patients were older and had a higher predicted risk of mortality. The rates of 30-day mortality, stroke, and readmission were similar in the 2 groups. ViVBVF achieved significantly greater reductions in mean gradient (−26.0 mm Hg vs −17.4 mm Hg; *P =*.003), peak gradient (−39.2 mm Hg vs −23.3 mm Hg; *P =*.004), and peak velocity. After matching, 30-day mortality was higher after redo-SAVR (8.9% vs 0%; *P* < .001). The ViVBVF group showed a greater reduction in peak velocity but more frequent pacemaker implantation.

**Conclusions:**

In patients with bioprosthetic valves requiring reintervention, ViVBVF offers greater gradient reduction, with a perioperative safety advantage suggested in the adjusted analysis.

**Figure:**
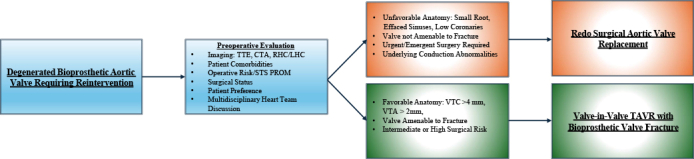
Management of degenerated bioprosthetic aortic valves requiring reintervention

**Central Message**

In patients with failed bioprosthetic aortic valves, valve-in-valve TAVR with bioprosthetic valve fracture provides superior hemodynamics and a perioperative safety advantage compared with redo SAVR.

**Perspective**

Reintervention for degenerated surgical aortic valves is increasingly common, yet the optimal strategy remains a topic of debate. This study provides the first direct comparison of redo-SAVR and valve-in-valve TAVR with bioprosthetic valve fracture, demonstrating improved hemodynamics and early safety with a less invasive approach.

Bioprosthetic valves have become the valve of choice for most patients, including younger individuals, to avoid the challenges associated with the lifelong anticoagulation required with the use of mechanical valves.^1^^,^^2^ However, bioprosthetic valves are subject to structural deterioration, necessitating reintervention within several decades.^3^^,^^4^ Traditionally, redo surgical aortic valve replacement (SAVR) was the sole available option for addressing a failed bioprosthetic aortic valve (AV), a procedure associated with increased operative risks owing to such factors as hostile chest reentry, aging patients, and worsening comorbidities.^5^^,^^6^ In recent years, valve-in-valve TAVR (ViV-TAVR) has emerged as a less invasive alternative to redo-SAVR, leveraging advances in transcatheter procedures to provide comparable hemodynamic results with reduced procedural complications and shorter recovery times.^7^^,^^8^

A significant limitation of ViV-TAVR is prosthesis–patient mismatch (PPM), which is especially prevalent in cases involving small-diameter surgical valves.^9^^,^^10^ The limited expansion of the transcatheter heart valve (THV) within the preexisting surgical valve frame constrains its effective orifice area (EOA) and may result in elevated transvalvular gradients, thereby reducing procedural efficacy and prosthesis durability.^2^^,^^11^ Bioprosthetic valve fracture (BVF) has been introduced as an off-label adjunct technique to address these limitations. By using noncompliant balloons inflated at high pressures, BVF fractures the rigid stent frame of certain bioprosthetic valves, enabling the implantation of a larger THV and thereby achieving improved hemodynamics.^9^^,^^10^ Early clinical series and bench studies demonstrated the feasibility of BVF, and more recent registry analyses confirm that it improves valve expansion, reduces gradients, and increases valve area at discharge.^12^ However, large-scale registry data also show that BVF is associated with higher rates of in-hospital mortality and life-threatening bleeding, underscoring that its hemodynamic benefits must be weighed against increased procedural risk.^12^ Moreover, not all bioprosthetic valves are amenable to fracture, and procedural success depends on valve type, timing (pre- or post-THV deployment), and operator expertise.^8^, ^9^, ^10^

Redo-SAVR, although more invasive, remains the standard for patients with high-risk features for ViV-TAVR, such as unfavorable coronary anatomy, small root or sinotubular junction anatomy, and valve types resistant to BVF.^8^^,^^9^ Nevertheless, redo surgery carries higher perioperative risk and is associated with prolonged recovery.^10^^,^^13^

To date, no comprehensive studies have directly compared the short-term clinical outcomes and echocardiographic results of patients with bioprosthetic structural valve deterioration undergoing redo-SAVR and ViV-TAVR with BVF (ViVBVF). This limited single-center retrospective analysis aims to offer early insight to guide patient selection, provide optimal treatment strategy management, and weigh potential advantages and complications of each intervention.

## Methods

Following Institutional Review Board approval (AAAV5230, approved November 6, 2024), data were collected retrospectively from the institutional registries and supplemented with chart review when appropriate. Informed consent was not required. The study included all patients diagnosed with a degenerated bioprosthetic AV who were considered for isolated bioprosthetic aortic valve replacement (AVR) at Columbia University Irving Medical Center between July 1, 2015, and January 1, 2025. Indications for AVR were determined based on preoperative clinical judgment and echocardiographic findings. A total of 128 consecutive patients, 75 with redo-SAVR and 53 with ViVBVF, were identified and included in the study. The ViVBVF cohort comprised 30 balloon-expandable and 23 self-expandable implanted valves.

### Study Population: Inclusion and Exclusion Criteria

Inclusion criteria were prior surgical bioprosthetic AVR, structural valve degeneration requiring reintervention, and treatment with either isolated redo-SAVR or ViV-TAVR with planned BVF. Exclusion criteria included active endocarditis, prior mechanical valve, requirement for concomitant cardiac procedure, and anatomic unsuitability for VIVBVF.

### Procedural Approach and Follow-up

Patients were stratified into 2 cohorts based on the reintervention performed, redo-SAVR or ViVBVF. Redo-SAVR was performed via median sternotomy in all patients. Standard cardiopulmonary bypass with ascending aortic cannulation and cross-clamping was used.

All patients in the ViVBVF group underwent preoperative gated computed tomography angiography. Procedures were performed via a transfemoral approach under moderate sedation or general anesthesia. A noncompliant balloon ≥1 size(s) larger than the surgical valve stent’s internal dimension was used to fracture the stent frame before deployment of a balloon- or self-expandable transcatheter valve. Patients with anatomic features unsafe for BVF (eg, valve-to-coronary distance <4 mm, valve-to-aorta distance <2 mm, sinuses equal to or <2 mm larger than the size of the THV, sinotubular junction diameter equal or smaller than the size of the implanted valve) were excluded from the study.

All patients underwent preoperative transthoracic echocardiography (TTE). Valve performance was assessed on predischarge TTE gradients. Clinical and echocardiographic data were collected retrospectively through chart review under Institutional Review Board approval.

### Endpoints

The primary endpoints were 30-day all-cause mortality, stroke, and valve-related readmission. Secondary endpoints included TTE-detected changes in AV mean and peak gradients, aortic peak velocity, degree of insufficiency, and ejection fraction. Additional secondary endpoints included the need for a permanent pacemaker and reintervention for bleeding.

### Statistical Analysis

All statistical analyses were performed in R version 4.4.2 (R Foundation for Statistical Computing). Continuous variables were summarized as mean ± standard deviation (SD) or median with interquartile range (IQR), based on the Shapiro-Wilk test of normality. Categorical variables were reported as count and percentage. Group comparisons were performed using the χ^2^ test or Fisher exact test for categorical variables and the 2-sample *t* test for normally distributed continuous variables or the Wilcoxon rank-sum test for non-normally distributed continuous variables. A *P* value < .05 was considered the threshold for statistical significance. Missing data were not imputed; instead, missing values were noted directly.

Spaghetti plots illustrated patient-level changes in AV mean gradient from preoperative to postoperative assessment, stratified by procedure type. Mean AV gradient change was categorized as decrease, increase, or no change.

Because the redo-SAVR and ViVBVF groups differed in baseline characteristics, propensity score (PS)–adjusted analysis using overlap weighting was performed to reduce confounding. PSs were estimated using logistic regression with prespecified covariates selected for clinical relevance and potential confounding: age, sex, prior coronary artery bypass grafting (CABG) or percutaneous coronary intervention, peripheral arterial disease, last (preprocedure) creatinine level, and Society of Thoracic Surgeons Predicted Risk of Mortality (STS-PROM). Overlap weighting targets the subset of patients in which the 2 treatments are both plausible. Weights are defined as w = 1 − PS for treated patients and w = PS for controls. This approach limits the impact of patients with extreme probabilities of receiving one treatment over the other and improves covariate balance while retaining the full study sample for analysis.

**Figure E1:**
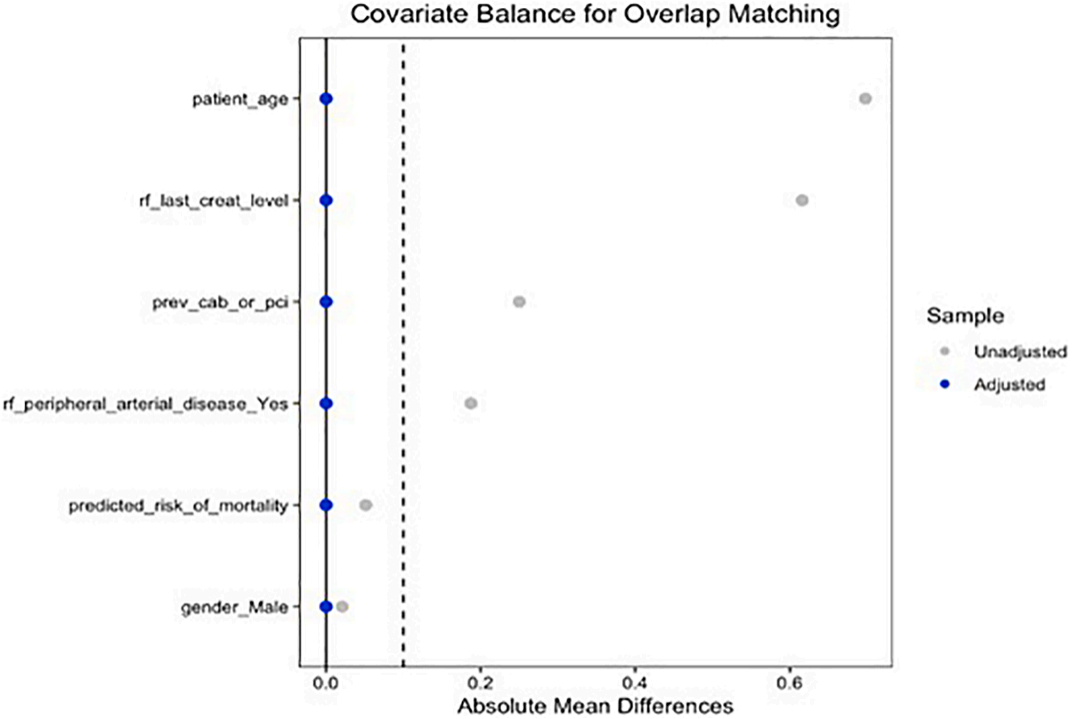
Covariate balance for overlap matching analysis.

Covariate balance after weighting was assessed using standardized mean difference (SMD), with an absolute SMD <0.1 considered indicative of adequate balance. Figure E1 summarizes preweighting and postweighting balance.

## Results

### Unadjusted Analysis

A total of 128 patients with a degenerated bioprosthetic AV were included in our study, of whom 75 (58.6%) underwent redo-SAVR and 53 (41.4%) underwent ViVBVF. Baseline and perioperative characteristics are summarized in Table 1. Patients in the ViVBVF group were older (median, 75 years; IQR, 66-79 years) compared with redo-SAVR patients (70 years; IQR, 64-74 years; *P =* .014). This cohort had a male predominance (65.6%). The STS-PROM was higher in the ViVBVF group compared with the redo-SAVR group (median, 3.63 [IQR, 1.84-4.57] vs 2.51 [IQR, 1.30-4.27]; *P* = .097).

**Table 1:** Unadjusted cohort analysis characteristics

Variable	Overall	Redo-SAVR	ViVBVF	*P* value
Number of patients	128	75	53	
Male sex, n (%)	84 (65.6)	48 (64.0)	36 (67.9)	.786
Patient age, y, median [IQR]	71.00 [64.00-76.00]	70.00 [64.00-74.00]	75.00 [66.00-79.00]	.014
Body surface area, mean (SD)	1.95 (0.26)	1.96 (0.27)	1.95 (0.24)	.878
Immunocompromised status, n (%)	10 (7.8)	3 (4.0)	7 (13.2)	.115
Prior CVA, n (%)	19 (14.8)	13 (17.3)	6 (11.3)	.49
History of chronic lung disease, n (%)	25 (19.5)	18 (24.0)	7 (13.2)	.197
History of diabetes, n (%)	30 (23.4)	14 (18.7)	16 (30.2)	.192
History of endocarditis, n (%)	32 (25.0)	28 (37.3)	4 (7.5)	<.001
History of hypertension, n (%)	107 (83.6)	58 (77.3)	49 (92.5)	.042
Prior MI, n (%)	10 (7.8)	5 (6.7)	5 (9.4)	.81
Peripheral artery disease, n (%)	16 (12.5)	4 (5.3)	12 (22.6)	.008
Previous CABG, n (%)	27 (21.1)	8 (10.7)	19 (35.8)	.001
Previous PCI, n (%)	23 (18.0)	9 (12.0)	14 (26.4)	.063
Preoperative creatinine, mg/dL, median [IQR]	1.02 [0.81-1.25]	1.00 [0.77-1.20]	1.09 [0.91-1.39]	.079
AV annular calcification, n (%)	55 (43.7)	36 (49.3)	19 (35.8)	.186
AV insufficiency severity, n (%)				.003
0 (none)	31 (24.8)	26 (35.6)	5 (9.6)	
1 (trace)	20 (16.0)	8 (11.0)	12 (23.1)	
2 (mild)	19 (15.2)	7 (9.6)	12 (23.1)	
3 (moderate)	19 (15.2)	9 (12.3)	10 (19.2)	
4 (severe)	36 (28.8)	23 (31.5)	13 (25.0)	
Aortic valve stenosis present, n (%)	122 (95.3)	69 (92.0)	53 (100.0)	.092
Preoperative AV mean gradient, median [IQR]	34.30 [22.20-43.53]	30.05 [15.60-40.08]	39.50 [30.00-48.50]	<.001
Preoperative AV peak gradient, median [IQR]	61.05 [39.75-76.25]	53.45 [27.00-69.92]	70.00 [55.50-83.00]	<.001
Preoperative AV peak velocity, median [IQR]	3.90 [3.15-4.30]	3.80 [2.65-4.20]	4.10 [3.50-4.40]	.01
Urgent/emergent status, n (%)	38 (29.7)	29 (38.7)	9 (17.0)	.014
STS-PROM, median [IQR]	2.80 [1.68, 4.44]	2.51 [1.30-4.27]	3.63 [1.84-4.57]	.097
Postoperative complication: stroke, n (%)	3 (2.3)	2 (2.7)	1 (1.9)	1
Postoperative complication: bleeding, n (%)	6 (4.7)	4 (5.3)	2 (3.8)	1
Need for PPM at 30 d, n (%)	14 (10.9)	5 (6.7)	9 (17.0)	.12
Postoperative AV peak gradient, median [IQR]	24.00 [16.25, 32.50]	23.30 [16.08-29.83]	24.70 [19.65-33.35]	.435
Postoperative AV mean gradient, median [IQR]	12.00 [8.10, 16.50]	11.90 [8.23-15.22]	13.00 [8.10-18.00]	.254
Postoperative AV peak velocity, median [IQR]	2.45 (0.53)	2.41 (0.50)	2.50 (0.58)	.367
Postoperative AV insufficiency severity, median [IQR]	0.00 [0.00, 0.00]	0.00 [0.00-0.00]	0.00 [0.00-0.00]	.459
AV peak gradient Δ, mean (SD)	−29.63 (29.31)	−23.29 (30.45)	−39.22 (24.88)	.004
AV mean gradient Δ, mean (SD)	−21.00 (15.22)	−17.35 (15.39)	−26.00 (13.60)	.003
AV peak velocity Δ, median [IQR]	−1.30 [−1.90, −0.40]	−1.20 [−1.75 to 0.20]	−1.60 [−2.10 to −0.65]	.019
AV insufficiency severity Δ, median [IQR]	−2.00 [−3.00, 0.00]	−2.00 [−3.00 to 0.00]	−2.00 [−3.00 to −1.00]	.448
Readmission within 30 d, n (%)				.082
No	115 (92.0)	67 (93.1)	48 (90.6)	
Unknown	5 (3.9)	5 (6.7)	0 (0.0)	
Yes	8 (6.2)	3 (4.0)	5 (9.4)	
Mortality status at 30 d, n (%)				.422
Alive	122 (95.3)	70 (93.3)	52 (98.1)	
Dead	5 (3.9)	5 (6.7)	1 (1.9)	
Preoperative LVEF, %, median [IQR]	55.00 [50.75-63.00]	55.00 [50.00-60.00]	58.00 [51.00-63.00]	.146
Postoperative LVEF, %, median [IQR]	55.00 [50.00-60.00]	55.00 [55.00-60.00]	58.00 [50.00-60.00]	.66
ΔLVEF, %, mean (SD)	−1.45 (10.22)	−0.86 (11.46)	−2.27 (8.22)	.453

*SAVR*, Surgical aortic valve replacement; *ViVBVF*, valve-in-valve transcatheter aortic valve replacement with bioprosthetic valve fracture; *IQR*, interquartile range; *SD*, standard deviation; *CVA*, cerebrovascular accident; *MI*, myocardial infarction; *CABG*, coronary artery bypass grafting; *PCI*, percutaneous coronary intervention; *AV*, aortic valve; *STS-PROM*, Society of Thoracic Surgeons Predicted Risk of Mortality; *PPM*, permanent pacemaker; *LVEF*, left ventricular ejection fraction.

Redo-SAVR patients had a higher rate of prior endocarditis (37.3% vs 7.5%; *P* < .001) and more commonly underwent urgent or emergent procedures (38.7% vs 17.0%; *P* = .014). Hypertension, peripheral arterial disease, and prior CABG were more frequent in the ViVBVF group (92.5% vs 77.3% [*P* = .042], 22.6% vs 5.3% [*P* = .008], and 35.8% vs 10.7% [*P* = .001], respectively).

Thirty-day mortality was not statistically different between the redo-SAVR and ViVBVF groups (6.7% vs 1.9%; *P* = .403). Stroke occurred infrequently in both groups (2.7% for redo-SAVR vs 1.9% for ViVBVF; *P* = 1.00). Readmissions within 30 days tended to be higher in the ViVBVF group (9.4% vs 4.0%; *P* = .082).

**Figure 1:**
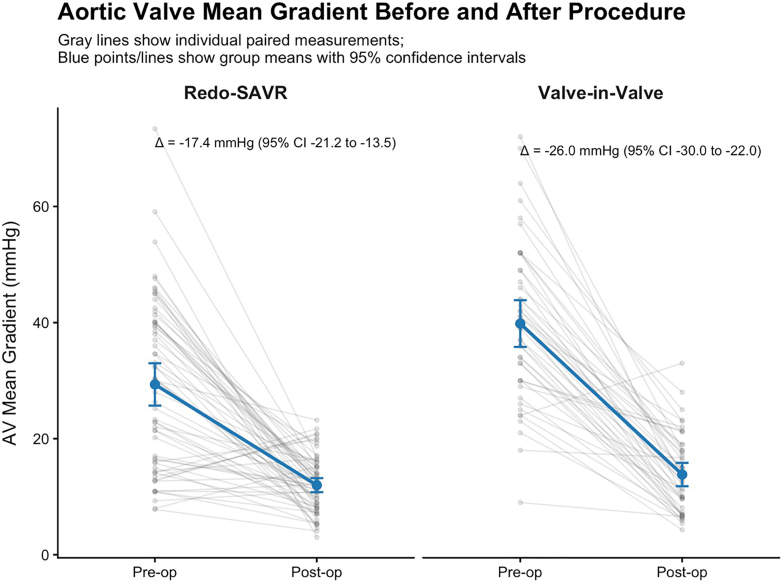
Spaghetti plot showing mean aortic valve gradient change in redo surgical aortic valve replacement (*SAVR*) versus valve-in-valve transcatheter aortic valve replacement with bioprosthetic valve fracture (*ViVBVF*).

Both groups demonstrated significant reductions in AV gradients postoperatively (Figure 1). The mean reduction in AV mean gradient was greater after ViVBVF compared with redo-SAVR (−26.0 ± 13.6 mm Hg vs −17.4 ± 15.4 mm Hg; *P* = .003). Similarly, the mean reduction in peak gradient were larger with ViVBVF (−39.2 ± 24.9 mm Hg vs −23.3 ± 30.5 mm Hg; *P* = .004), as was the median decrease in peak velocity (median −1.60 [IQR, −2.10 to −0.65] m/s vs −1.20 [IQR, −1.75 to 0.20] m/s; *P* = .019). Postoperative mean gradients and peak velocities were otherwise similar in the 2 groups.

The need for permanent pacemaker implantation at 30 days was higher in the ViVBVF group (17.0% vs 6.7% *P* = .120). Major vascular complications necessitating reintervention were similar in the 2 groups (5.3% for redo-SAVR vs 3.8% for ViVBVF; *P* = 1).

### Adjusted Analysis

In the overlap-adjusted analysis, 18 patients undergoing redo-SAVR were compared with 18 patients undergoing ViVBVF (Table 2). The 2 groups were adjusted for sex, age, history of peripheral arterial disease, prior CABG or percutaneous coronary intervention, and STS-PROM to minimize confounding (Figure E1).

**Table 2:** Adjusted cohort analysis characteristics

Variable	Redo-SAVR	ViVBVF	*P* value
Number of patients	18	18	
Male sex, n (%)	11 (62.7)	11 (62.7)	1
Patient age, y, median [IQR]	69 (8.76)	69 (10.01)	1
Body surface area, mean (SD)	1.92 (0.28)	1.93 (0.26)	.994
Immunocompromised status, n (%)	0 (2.3)	1 (6.8)	.22
Prior CVA, n (%)	2 (11.4)	2 (9.7)	.817
History of chronic lung disease, n (%)	5 (25.4)	2 (9.9)	.068
History of diabetes, n (%)	4 (20.7)	5 (27.3)	.513
History of hypertension, n (%)	15 (80.7)	16 (87.8)	.426
Prior MI, n (%)	2 (9.1)	1 (4.9)	.437
Peripheral artery disease, n (%)	2 (8.1)	2 (8.1)	1
Previous CABG, n (%)	4 (23.3)	4 (23.3)	1
Preoperative creatinine, mg/dL, median [IQR]	1.01 (0.28)	1.01 (0.32)	1
AV annular calcification, n (%)	9.4 (52.4)	6.5 (36.1)	.178
Aortic valve stenosis present, n (%)	17.5 (96.9)	18.1 (100.0)	.177
Urgent/emergent status, n (%)	2.9 (15.8)	2.1 (11.4)	.534
STS-PROM, median [IQR]	4.04 (11.27)	4.04 (4.43)	1
Postoperative complication: stroke, n (%)	0.3 (1.5)	0.4 (2.1)	.823
Postoperative complication: bleeding, n (%)	1.6 (9.0)	0.4 (2.1)	.152
Need for permanent pacemaker at 30 d, n (%)	0.7 (3.8)	2.2 (12.4)	.241
AV mean gradient Δ, median [IQR]	−17.11 (17.90)	−24.74 (17.81)	.096
Preoperative AV peak gradient, median [IQR]	59.22 (26.13)	70.82 (23.68)	.08
Postoperative AV peak gradient, median [IQR]	24.29 (8.24)	27.84 (12.88)	.164
AV peak gradient Δ, median [IQR]	−26.34 (32.06)	−39.61 (28.02)	.088
Preoperative AV peak velocity, median [IQR]	3.74 (0.90)	4.02 (0.74)	.196
Postoperative AV peak velocity, median [IQR]	2.42 (0.45)	2.58 (0.60)	.19
AV peak velocity severity, median [IQR]	−0.68 (1.67)	−1.39 (0.86)	.032
Preoperative AV insufficiency, median [IQR]	2.04 (1.69)	2.02 (1.25)	.953
Postoperative AV insufficiency, median [IQR]	0.18 (0.48)	0.28 (0.77)	.498
AV insufficiency Δ, median [IQR]	−1.83 (1.88)	−1.70 (1.32)	.739
Readmission within 30 d, n (%)			.161
No	16 (92.5)	17 (95.3)	
Unknown	1 (6.3)	0.0 (0.0)	
Yes	0 (1.2)	1 (4.7)	
Mortality status at 30 d, n (%)			<.001
Alive	17 (91.1)	18 (99.8)	
Dead	2 (8.9)	0.0 (0.2)	

*SAVR*, Surgical aortic valve replacement; *ViVBVF*, valve-in-valve transcatheter aortic valve replacement with bioprosthetic valve fracture; *IQR*, interquartile range; *SD*, standard deviation; *CVA*, cerebrovascular accident; *MI*, myocardial infarction; *CABG*, coronary artery bypass grafting; *AV*, aortic valve; *STS-PROM*, Society of Thoracic Surgeons Predicted Risk of Mortality.

Thirty-day mortality differed significantly between redo-SAVR and ViVBVF (8.9% vs 0.2%; *P*< .001). Stroke rates were similar in the 2 groups (1.5% vs 2.1%; *P*= .823). A higher percentage of ViVBVF recipients were readmitted after discharge (40.7% vs 1.2%; *P*= .161).

Postoperative peak gradients were similar in the 2 groups (mean, 27.8 ± 12.9 mm Hg for ViVBVF vs 24.3 ± 8.2 mm Hg for redo-SAVR; *P* = .164) and the delta values in peak gradient and velocity tended to favor ViVBVF (−39.6 ± 28.0 vs −26.3 ± 32.1 [*P* = .088] and −1.39 ± 0.86 m/s vs −0.68 ± 1.67 m/s [*P* = .032], respectively).

More patients in the ViVBVF group required permanent pacemaker implantation at 30 days (12.4% vs 3.8%; *P* = .241). Reoperation for bleeding was more common in the redo-SAVR group (9.0% vs 2.1%; *P* = .152). The rate of readmission was 1.2% in the redo-SAVR group and 4.7% in the ViVBVF group (*P* = .161).

## Discussion

This single-center retrospective study comparing the short-term clinical and echocardiographic outcomes of patients undergoing AV reinterventions, specifically redo-SAVR and ViVBVF, revealed the following meaningful findings: (1) early clinical outcomes were favorable in both groups, with low 30-day mortality and stroke rates; (2) redo-SAVR carried a significantly higher 30-day mortality when groups were adjusted; (3) ViVBVF achieved greater improvement in valve hemodynamics; (4) ViVBVF patients were more likely to require a permanent pacemaker; and (5) hospital readmissions were less frequent after redo-SAVR. Both redo-SAVR and ViVBVF are valuable and well-established options for the treatment of degenerated aortic bioprostheses. Although redo-SAVR is more invasive and carries higher periprocedural risks, it remains a widely adopted solution offering good long-term outcomes.^13^ ViV- TAVR was introduced in 2015 with the aim of limiting the challenges of redo sternotomy and providing a faster, easier, and equally efficient option for treating a failed bioprosthesis.^3^^,^^7^

One limitation of ViV-TAVR is the poor hemodynamic profile of the TAVR, especially when implanted in a small-sized bioprosthesis. The suboptimal expansion of the THV owing to the rigid sewing cuff may result in some degree of PPM and higher postoperative gradients. A recent meta-analysis of nearly 82,000 patients demonstrated that severe PPM after TAVR, although less common than after SAVR, was associated with a significantly increased risk of mortality.^14^ Complementing these findings, in vivo imaging studies have shown that THV underexpansion and deformation are common after ViV-TAVR, and that these geometric constraints directly correlate with higher gradients and smaller EOA.^12^

While surgeons opt for root enlargement to implant a larger-sized aortic prosthesis, a way to obviate PPM in the ViV-TAVR procedure is to perform BVF.^11^ For this, a noncompliant balloon is inflated at high-pressure inside the surgical valve, and the metal stent is “fracked,” allowing implantation of a larger THV. BVF remains an off-label intervention that carries intrinsic risks, and it can be associated with major life-threatening complications, including annular rupture, stroke, and death. Redo-SAVR and ViV-TAVR have been widely studied and compared in multiple registries.^4^^,^^7^^,^^9^^,^^10^^,^^13^^,^^15^ The adjunct of BVF to ViV-TAVR compared to redo-SAVR has not been subject to significant investigation, however.

Our present analysis aimed to understand whether adding valve fracture to the ViV-TAVR intervention provided any hemodynamic and clinical short-term benefit compared to redo-SAVR. Our results demonstrate that both procedures can be performed with acceptable perioperative risk. While 30-day mortality was low in both analyses, the significant mortality difference in the adjusted analysis does suggest a potential perioperative signal favoring VIVBVF, although given the limited effective sample size, this finding should be interpreted cautiously. Despite comparable procedural success, early deaths in the redo-SAVR group were primarily attributable to the intrinsic hazards of reoperative cardiac surgery (Table E1).

Our findings are consistent in both the unadjusted and adjusted populations; however, the significantly higher 30-day adjusted mortality in redo-SAVR patients reflects the well-recognized perioperative hazards of repeat sternotomy, including bleeding, arrhythmia, and organ dysfunction.^7^ This suggests that while both procedures are safe in appropriately selected patients, ViVBVF may provide a perioperative safety advantage.

A consistent finding across both analyses was the superior reduction in gradients achieved with ViVBVF. In the unadjusted cohort, ViVBVF patients demonstrated larger decreases in mean gradient, peak gradient, and peak velocity. Similar trends were observed in the matched cohort. These findings are consistent with prior reports demonstrating that BVF optimizes THV expansion and improves EOA.^1^^,^^11^ Clinical series and registry data confirm that BVF reliably lowers residual gradients, particularly in small surgical valves.^9^ Our study reinforces these observations, showing that the hemodynamic benefit of BVF is robust even after risk adjustment.

Residual gradients after ViVBVF largely reflect PPM, a major determinant of outcomes. Severe PPM, defined by an indexed EOA (iEOA) <0.65 cm^2^/m^2^, has been associated with increased mortality after both SAVR and TAVR.^16^ Fukui and colleagues demonstrated that suboptimal THV expansion is a key contributor to PPM.^8^ BVF directly addresses this by fracturing the surgical valve, permitting more complete THV expansion.^1^^,^^11^ In our study, the larger gradient delta value observed with BVF is consistent with this mechanism. However, a limitation to our analysis was the absence of iEOA data, which prevented direct assessment of PPM incidence. The postprocedural valve performance was based solely on crude echocardiographic measurements. We hypothesize that BVF mitigates clinically significant PPM. Whether this translates into long-term survival benefit has yet to be demonstrated. In those patients with a small bioprosthesis and some degree of PPM at baseline, redo-SAVR remains the sole available treatment option.

While early and mid-term outcomes favor ViVBVF for hemodynamic performance, the long-term durability of this strategy remains uncertain. Observational studies have shown that although ViVBVF provides lower early risk, it may be associated with higher late mortality.^3^ Patel and colleagues^7^ also reported higher reintervention rates after ViVBVF but more durable valve performance after redo-SAVR. Another aspect to consider is the timing of BVF in ViV-TAVR cases. We always performed the valve fracture before the TAVR implantation, to avoid exposure of the fresh leaflets to high-pressure balloon inflations. Compared to fracturing the surgical valve after the THV implantation, this strategy has been associated with increased mortality and minimal changes in hemodynamic parameters.^12^ Nevertheless, our study shows that performing BVF first does not change the overall risk of the procedure and provides a beneficial reduction of transvalvular gradients. Long-term clinical and echocardiographic follow-up data are missing, however, leaving the effect of the high-pressure balloons on the THV questionable.

A notable finding from our analysis was that only 18 patients per group could be adjusted successfully despite the use of overlap weighting. This reflects the significant differences between the redo-SAVR and ViVBVF cohorts. Patients undergoing redo-SAVR were more likely to present urgently or emergently and had a higher rate of history of endocarditis, whereas ViVBVF patients generally were older with more comorbidities. These distinctions highlight that redo-SAVR and ViVBVF are often offered to fundamentally different patient populations, which complicates direct comparison. While matching helps mitigate confounding, the small, matched analysis underscores the challenges of drawing definitive conclusions and differences in postoperative complications, including vascular events and readmissions, should be interpreted with caution. It also reinforces the notion that a personalized approach to AV reintervention is critical and that there are 2 effective possible interventions available depending on patient presentation, anatomy, and comorbidities.

This study focused on short-term outcomes and does not address intermediate or long-term valve durability. Ongoing follow-up within our institutional registry will be necessary to determine whether the early hemodynamic advantages observed with VIVBVF translate into improved longer-term outcomes.

## Conclusions

We found that ViVBVF offers superior gradient reduction and comparable early outcomes to redo-SAVR, with a perioperative safety advantage suggested in adjusted analysis. These findings underscore the complementary roles of each strategy: redo-SAVR as a durable but higher-risk option and ViVBVF as a less invasive approach for high-risk patients. Future studies will be essential to determine whether the hemodynamic benefits of ViVBVF translate into improved long-term outcomes compared to redo-SAVR.

## Conflict of Interest Statement

The authors reported no conflicts of interest.

The *Journal* policy requires editors and reviewers to disclose conflicts of interest and to decline handling or reviewing manuscripts for which they may have a conflict of interest. The editors and reviewers of this article have no conflicts of interest.

## Figures and Tables

**Table E1:** Timing and etiology of mortality following redo-SAVR and ViVBVF

Cohort	Deaths, n	Timing of death	Cause of death
Redo-SAVR	1	Intraoperative	Intraoperative cardiac arrest
Redo-SAVR	3	Postdischarge	Survived to discharge but died of unknown causes
Redo-SAVR	1	Early postoperative	High-risk with unfavorable anatomy for ViVBVF resulting in multiorgan failure and death in the ICU
ViVBVF	1	Early postoperative	PEA arrest during unsuccessful BVF requiring VA-ECMO, with subsequent death in the ICU

*SAVR*, Surgical aortic valve replacement; *ViVBVF*, valve-in-valve transcatheter aortic valve replacement with bioprosthetic valve fracture; *ICU*, intensive care unit; *PEA*, pulseless electrical activity; *BVF*, bioprosthetic valve fracture; *VA-ECMO*, venoarterial extracorporeal membrane oxygenation.
